# Evoked directional network characteristics of epileptogenic tissue derived from single pulse electrical stimulation

**DOI:** 10.1002/hbm.24309

**Published:** 2018-07-21

**Authors:** Dorien van Blooijs, Frans S. S. Leijten, Peter C. van Rijen, Hil G. E. Meijer, Geertjan J. M. Huiskamp

**Affiliations:** ^1^ Department of Neurology and Neurosurgery, Brain Center Rudolf Magnus University Medical Center Utrecht Utrecht The Netherlands; ^2^ Department of Applied Mathematics MIRA Institute for Biomedical Engineering and Technical Medicine, University of Twente Enschede The Netherlands

**Keywords:** bidirectional network, electrocorticography, epilepsy, epileptogenic zone, single pulse electrical stimulation, surgery

## Abstract

We investigated effective networks constructed from single pulse electrical stimulation (SPES) in epilepsy patients who underwent intracranial electrocorticography. Using graph analysis, we compared network characteristics of tissue within and outside the epileptogenic area. In 21 patients with subdural electrode grids (1 cm interelectrode distance), we constructed a binary, directional network derived from SPES early responses (<100 ms). We calculated in‐degree, out‐degree, betweenness centrality, the percentage of bidirectional, receiving and activating connections, and the percentage of connections toward the (non‐)epileptogenic tissue for each node in the network. We analyzed whether these network measures were significantly different in seizure onset zone (SOZ)‐electrodes compared to non‐SOZ electrodes, in resected area (RA)‐electrodes compared to non‐RA electrodes, and in seizure free compared to not seizure‐free patients. Electrodes in the SOZ/RA showed significantly higher values for in‐degree and out‐degree, both at group level, and at patient level, and more so in seizure‐free patients. These differences were not observed for betweenness centrality. There were also more bidirectional and fewer receiving connections in the SOZ/RA in seizure‐free patients. It appears that the SOZ/RA is densely connected with itself, with only little input arriving from non‐SOZ/non‐RA electrodes. These results suggest that meso‐scale effective network measures are different in epileptogenic compared to normal brain tissue. Local connections within the SOZ/RA are increased and the SOZ/RA is relatively isolated from the surrounding cortex. This offers the prospect of enhanced prediction of epilepsy‐prone brain areas using SPES.

## 1 INTRODUCTION

Epilepsy surgery is a highly effective therapy in selected people with focal epilepsy. In patients without a clear lesion on MRI, or with a lesion potentially overlapping with eloquent cortex, chronic intracranial electrocorticography (ECoG) monitoring may be necessary to delineate the seizure onset zone (SOZ). The SOZ is defined as the region from which epileptic seizures arise, and is assumed to be an important part of the epileptogenic zone, removal of which should stop seizures (Lüders, 2008). Ictal ECoG provides the gold standard for localizing this SOZ which is characterized by a recruiting seizure rhythm preceding or coinciding with the first clinical signs of a seizure. Waiting for spontaneous seizures usually determines the length of the monitoring period, and may require days to weeks, with stress for the patient and risks of complications like intracranial infections or hemorrhage.

Single pulse electrical stimulation (SPES) is a clinical method for identifying the epileptogenic zone independent of spontaneous seizures, mainly because of the ability to provoke delayed responses (DRs) (Valentín et al., 2002; van ‘t Klooster et al., 2011). During the SPES protocol, electrocortical stimuli are systematically applied to pairs of adjacent electrodes on the subdural electrode grid and correlated responses in all other electrodes are analyzed. SPES can thus be used to reveal the physiological connections of cortical patches underlying the grid‐electrodes and has the potential to contribute to our understanding of the network basis of epilepsy on a mesoscale (Alarcón, Valentín, Alarcon, & Valentin, 2012). Within 100 ms after the stimulus, early responses (ERs) may be observed after SPES, suggesting physiological connections from cortex under the stimulated electrode pair to cortex under the electrodes in which ERs are observed (Alarcón et al., 2012). ERs occur each time a pulse is applied to the same electrode pair, and are thus deterministic. In cortico‐cortical evoked potential studies, this ER is known as the N1‐response (Enatsu, Piao, et al., 2012; Keller et al., 2014; Matsumoto et al., 2004, 2007) and the physiological networks derived from these N1‐responses have been investigated in, for example, the language and motor system (Matsumoto et al., 2004, 2007).

Physiological networks may be altered in brain diseases like epilepsy. Over the last decade, the concept of focal epilepsy as a localized region of abnormality has evolved into a concept of diseased cortical networks with nodes and connections also affected in regions away from the SOZ (Antony et al., 2013; Bartolomei et al., 2017; Bernhardt, Bonilha, & Gross, 2015; Spencer, 2002; van Diessen, Diederen, Braun, Jansen, & Stam, 2013; van Diessen et al., 2014). In the context of epilepsy surgery, the focus on defining only a local SOZ is disputed, as the whole brain network operates together, as is clear from the expression of seizures (Spencer, 2002). It has been suggested that seizure freedom may be best achieved by removing a critical part of tissue that interrupts the epileptic network (Hebbink, Meijer, Huiskamp, van Gils, & Leijten, 2017).

In epilepsy research, networks have been reconstructed with data from fMRI, DTI, MEG, EEG, or intracranial EEG, from ictal, pre‐ictal, or interictal periods, at different scales and with different methodological approaches. A network consists of nodes and edges. Nodes represent functional or structural elements of the network (van Diessen, et al., 2013), or in case of SPES, a cortical patch underneath an ECoG electrode. Edges represent a connection between two areas.

Networks can be categorized as anatomical, functional, or effective networks. Anatomical networks are derived from structural axonal bundles between different brain regions (Yaffe et al., 2015). Functional networks assess connectivity based on statistical dependencies between neuronal activity at different locations. Effective networks describe the causal interactions between neural elements caused by perturbation experiments like stimulation or SPES.

With graph analysis, the overall network characteristics can be quantified. Examples of commonly used graph measures are degree and betweenness centrality. The degree of a node is equal to the number of edges connected to that node. This value reflects the importance of an individual node in the network. The degree has a straightforward neurobiological interpretation: nodes with a high degree interact with many other nodes in the network. The degree can be directional and characterized as in‐ and out‐degree; that is, the number of incoming connections, or outgoing connections, respectively (van Mierlo, Papadopoulou, et al., 2013).

The betweenness centrality is defined as the fraction of all shortest paths between nodes in the network that pass through a given node (Rubinov & Sporns, 2010; van Diessen, Hanemaaijer, et al., 2013). Nodes connecting different parts of the network often have a high betweenness centrality (Rubinov & Sporns, 2010). In other words, betweenness centrality is a measure of the “importance” of a node to transfer information across the network. Unlike other measures that quantify network properties for a node, the betweenness centrality depends not only on the primary efferent and afferent connections to a node, but also on the secondary and tertiary connections (Wilke, Worrell, & He, 2011).

Both for functional and anatomical networks, graph analysis have been applied extensively in epilepsy research. For instance, van Mierlo, Carrette, et al. (2013) constructed a directed functional connectivity graph during seizure onset in intracerebral EEG using the adaptive directed transfer function, from which they concluded that the electrode with the highest out‐degree coincided best with the SOZ. Van Diessen, Otte, Stam, Braun, and Jansen (2016) demonstrated in scalp EEG‐data that interictal network alterations are present in epilepsy patients. They showed that the betweenness centrality was overall significantly lower in networks of children with focal epilepsies compared to healthy children.

Analysis of SPES networks has revealed information by location and amplitude of evoked ERs. Mouthaan et al. (2015) found high counts of ERs in the SOZ. Enatsu, Jin, et al. (2012) showed that the amplitude of ERs in and outside the SOZ was higher when a stimulus was applied within the SOZ. Boido et al. (2014) categorized electrodes as “activator,” “receiver,” or “bidirectional contact” based on the number of evoked ERs in and by each electrode. Activators were electrodes with many outgoing connections, receivers were electrodes with many ingoing connections, and a bidirectional contact had many in‐ and out‐going connections. They found a significant association between bidirectional electrodes and the SOZ.

So far, SPES networks have shown that location and amplitude are important measures for distinguishing the epileptogenic tissue, but this has not been analyzed in terms of network measures. In the present study, we combine analysis of the SPES network and the common network measures in‐degree, out‐degree, and betweenness centrality to investigate the properties of epileptogenic tissue using the SPES network. Furthermore, we analyze the directionality of connections (Boido et al., 2014), and the destination of connections. Specifically, we investigate whether network characteristics are different in presumed epileptogenic tissue. We, therefore, constructed effective networks based on SPES ERs recorded during interictal periods collected in patients with focal epilepsy undergoing presurgical evaluation.

## 2 MATERIALS AND METHODS

### 2.1 Patients

We included patients who underwent long‐term clinical ECoG monitoring preceding epilepsy surgery between 2014 and 2016 in whom SPES was routinely performed for clinical decision making with stimuli applied in at least 90% of the electrodes. Patients who did not undergo resection were excluded. There was no overlap with patients included in previous studies by our group (Mouthaan et al., 2015; van ‘t Klooster et al., 2011). Patients had been admitted to the Intensive Epilepsy Monitoring Unit of the University Medical Centre of Utrecht, the Netherlands. All patients gave their informed consent and the entire investigation was performed under the ethical committee's approval under Dutch law.

### 2.2 Electrocorticography

Chronic ECoG was performed with subdural electrode grids (2–8 × 8) and strips (1 × 8 electrodes) placed directly on the cortex. They consisted of platinum circular electrodes embedded in silicone that had a 4.2 mm^2^ contact surface and an interelectrode distance of 1 cm. In four patients, also depth electrodes were implanted consisting of six cylindrical contacts with 7.9 mm^2^ contact surface at a 5 mm interelectrode distance (Ad‐Tech, Racine, WI).

### 2.3 Seizure onset zone and resected area

Two neurologists (CF, FL) localized the SOZ and projected the resected area (RA) on the grids in each patient. The SOZ was considered as the site with the earliest ictal activity, defined as patterns consisting of rhythmic spikes, sharp waves, spike and slow wave complexes, or recruiting gamma or beta activity. The RA usually contained the SOZ, but was sometimes larger because it included a lesion. Therefore, we used both areas as gold standards. We realize that we mentioned in the Section 1 that the concept of epilepsy as a network disease has evolved, and still define a particular epileptogenic area.

### 2.4 Single pulse data acquisition

SPES was performed during ECoG monitoring with ECoG data sampled at 2048 Hz to enable visualization of evoked activity up to 500 Hz (van ‘t Klooster et al., 2011) using a MicroMed LTM64/128 express EEG headbox with integrated programmable stimulator (MicroMed, Mogliano—Veneto, Italy). Ten monophasic stimuli of 1 ms pulse width were applied at a frequency of 0.2 Hz to two adjacent electrodes. A current intensity of 8 mA was used, but in case of twitches or pain, the intensity was lowered to 4 mA. SPES results were taken from the total number of electrode pairs (#trials) to which 10 pulses were applied. Results from clinical SPES and evoked DRs were used for the final clinical decision making in individual patients (van ‘t Klooster et al., 2011).

### 2.5 Analysis of early responses

**Figure 1:**
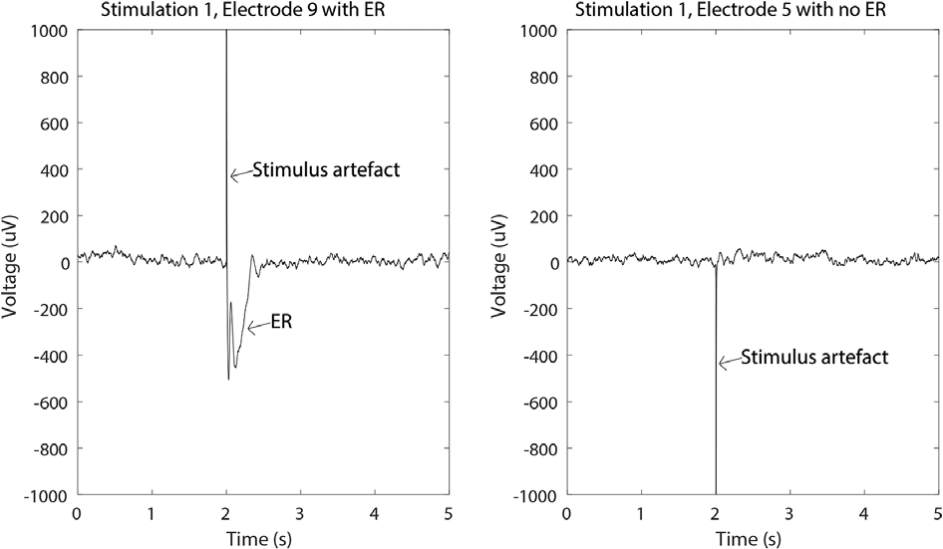
Visual check of epochs in which an ER was detected. Ten epochs were averaged, resulting in one signal for each stimulus pair‐response electrode combination. The left figure shows an averaged response in electrode 9 to stimulation of electrode pair 1–2. The straight line is the stimulus artifact, the ensuing negative wave of the ER. The right figure shows an averaged response in electrode 5 to stimulating the same electrode pair. No ER is observed

For each electrode, 10 epochs with a time window of 2 s prestimulus to 3 s poststimulus, time locked to the stimulus, were averaged for each trial. Each epoch was corrected for baseline (a time window of 2 s prior to stimulation). ERs were determined with an automatic detector in each averaged epoch. ERs were detected within 9–100 ms, when a peak exceeded the threshold of 2.5 times the standard deviation measured during baseline (Figure 1). The detected ERs were visually checked (DvB). Electrodes which overlapped with another grid, or were noisy, were not stimulated and therefore excluded from analysis.

### 2.6 Constructing a nodal network

In traditional functional networks, each electrode is represented by one node of the network (Burns et al., 2014). As stimuli in SPES are applied to stimulus pairs, such nodes had to be defined differently. ERs originate from stimulus pairs (two electrodes), and are observed in single electrodes. We adapted the SPES‐network to define a nodal network.

When a stimulus pair evoked an ER in another electrode, both electrodes in the stimulus pair were assumed to project onto the electrode in which an ER was observed.

#### 2.6.1 Out‐degree

**Figure 2:**
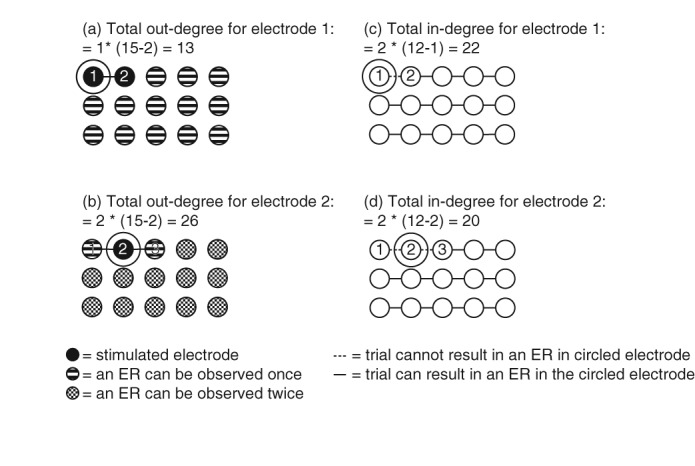
The difference in total out‐ and in‐degree for electrodes stimulated once or twice. Out‐degree: (a) a stimulus is applied only once to electrode 1 (electrode‐pair 1–2). In all other electrodes, an ER can be evoked only once. The maximal out‐degree is the total number of electrodes minus the 2 electrodes in the stimulus pair, resulting in a maximal out‐degree of 13. Indegree: (b) a total number of 12 trials with different stimulation pairs are applied in this example. Each trial can evoke an ER in a response electrode. In electrode 1, only one trial is applied. This results in a maximal in‐degree of two times the total number of trials minus the trials evolving the response electrode, resulting in a maximal in‐degree of 22. (c) Electrode 2 is stimulated twice (as part of electrode‐pairs 1–2 and 2–3). Therefore, the maximal out‐degree is two times the total number of electrodes minus the two electrodes in the stimulus pair, resulting in a maximal out‐degree of 26. (d) In electrode 2, two trials are applied, resulting in a maximal in‐degree of 20

Some electrodes were part of one stimulus pair, while others were part of two pairs. For example: electrode 1 was involved only in stimulus pair 1–2, whereas electrode 2 was involved in both 1–2 and 2–3. In electrode 1, the odds of detecting connections to other electrodes is half the chance of detecting connections to other electrodes in electrode 2 (Figure 2). Therefore, we normalized the number of ERs evoked by stimulating a specific electrode (out‐degree: *e*_ER→_) by dividing it by the maximal possible outgoing connections (*n*_out_total_), defined as: the number of trials in which the specific electrode is stimulated (*t*_e_) multiplied by the total number of potential response electrodes (*e*_tot_) minus 2 (the number of electrodes in a stimulus pair) (Equation 1).

#### 2.6.2 In‐degree

When a stimulus is applied to an electrode, no ER can be detected in this electrode. Therefore, in an electrode stimulated once, an ER can be observed in one more trial, compared to electrodes stimulated twice. For example: an ER cannot be observed in electrode 1 only when stimulating 1–2, whereas an ER cannot be observed in electrode 2 when stimulating 1–2 and 2–3 (Figure 2). We normalized the number of ERs evoked in a specific electrode (in‐degree: *e*_→ER_) by dividing it by the maximum possible incoming connections (*n*_in_total_), defined as: 2 (the number of electrodes in a stimulus pair), multiplied by the total number of trials (*t*_tot_) minus the number of trials in which the specific electrode was stimulated (*t*_e_) (Equation 2).

#### 2.6.3 Betweenness centrality

We normalized the betweenness centrality in each electrode (BC_e_) (Equation 3) by dividing it by the maximum number of incoming connections (*n*_in_total_) and the maximum number of outgoing connections (*n*_out_total_) as defined previously.

The modified measures are given by the following equations. They range between 0 and 1, where 0 meant that no connections were observed and 1 meant that all possible connections were observed.

Equation 1: out‐degree of node

$$n_{\text{outdegree}}=\frac{e_{\mathrm{ER}\to }}{t_{e}\;\left(e_{\mathrm{tot}}-2\right)}$$

Equation 2: in‐degree of node

$$n_{\text{indegree}}=\frac{e_{\to \mathrm{ER}}}{2\left(t_{\mathrm{tot}}-t_{e}\right)}$$

Equation 3: betweenness centrality in node

$$n_{\mathrm{BC}}=\frac{{\mathrm{BC}}_{e}}{n_{\text{in}\_\text{total}}n_{\text{out}\_\text{total}}}=\frac{{\mathrm{BC}}_{e}}{\left(2\left(t_{\mathrm{tot}}-t_{e}\right)\right)\left(\;t_{e}\left(e_{\mathrm{tot}}-2\right)\right)}$$

#### 2.6.4 Network measures in (non‐)SOZ and (non‐)RA

Per patient, we divided the electrodes into two groups: SOZ and non‐SOZ electrodes, RA and non‐RA electrodes. We determined whether differences in network measures between those regions were statistically significant (*p* < .05) using a Mann–Whitney *U* test.

We repeated the Mann–Whitney *U* test to determine statistical differences between the same groups (*p* < .05) over all patients, and also for patients with Engel I and patients who were not seizure free after surgery.

#### 2.6.5 Directionality of connections in each node

After interpretation of the results from the first analysis, we proceeded in studying the directionality of connections.

We classified these connections into bidirectional, activating (connections toward other nodes), and receiving (connections from other nodes) (Boido et al., 2014). Per patient, overall patients, and in seizure‐free or not seizure‐free patients, we determined whether there was a difference in directionality in SOZ‐ and non‐SOZ nodes, and in RA‐ and non‐RA nodes using a Mann–Whitney *U* test.

Then, we looked at the destination of connections from in and outside RA/SOZ. We calculated the ratio of connections from a specific node to the (non‐)RA from the total number of outgoing connections involving each specific node. We compared the ratio of connections from the (non‐)RA to both the RA nodes and non‐RA nodes using a Mann–Whitney *U* test. We repeated this test for the SOZ nodes and in patients with Engel I and patients who were not seizure free after surgery.

## 3 RESULTS

### 3.1 Patient characteristics

In total, 26 patients underwent grid monitoring between January 2014 and March 2016 (Table 1). Three patients did not undergo epilepsy surgery, because the SOZ could not be determined. Two patients were excluded because less than 90% of the electrodes were stimulated with SPES. Thus, 21 patients (11 females, 10 males), with a median age of 15 years (range: 4–49 years) were included. Six patients were not seizure free; 15 patients were seizure free after 1 year (Engel class Ia or Ib). ECoG involved a median number of 64 stimulated electrodes per patient (range: 48–86). The SOZ and RA were covered by a median number of 4.5 electrodes (range: 1–16) and 12 electrodes (range: 3–28), respectively. In each patient, a median number of 55 trials (range: 44–73) was applied.

**Table 1:** Patient characteristics

Patient #	Age	Sex	Grid location	#Electrodes	#Trials	#Electrodes SOZ	#Electrodes RA	Seizure free?
1	6	Fe	T, Oc	54	46	1	13	Y
2	10	Fe	F, C, D	66	55	ND	13	Y
3	15	M	T, P, Oc	54	45	8*	13	Y
4	42	Fe	T, P, Oc	75	64	4	22	Y
5	4	M	P, IH	55	47	3	9	Y
6	15	Fe	F, C	63	55	12*	6	Y
7	19	M	T, Oc	77	64	10*	13	N
8	25	Fe	T, P	70	60	ND	3	N
9	12	Fe	C, IH, D	48	40	10	12	Y
10	9	Fe	F, T, IH, C	80	70	16	19	Y
11	16	M	F, T, Oc, D	64	54	0	1	Y
12	49	M	C, T, P, Oc	67	62	7	9	Y
13	11	M	C	62	54	5*	9	N
14	13	M	P, C, IH, D	61	53	4*	16	Y
15	41	Fe	T	51	44	3	11	Y
16	14	Fe	F, C, T, IH	86	73	ND	15	Y
17	8	Fe	T, P, Oc	79	66	ND	28	N
18	18	M	C, IH	64	56	3	9	Y
19	15	Fe	C	60	51	2	10	Y
20	10	M	F, T, P	77	65	7*	10	N
21	19	M	T, F	62	51	ND	16	N

In the number of electrodes, electrodes SOZ/RA, only stimulated electrodes are included, * indicates the patients where SOZ was not completely resected. ND = not determined. In five patients, the SOZ could not be delineated due to diffuse seizure onset (patients 2, 8, 16, and 21) or status epilepticus during monitoring period (patient 17). Resection was then based on the location of a lesion on MRI. M = male; Fe = female; F = frontal; C = central; T = temporal; P = parietal; Oc = occipital; IH = interhemispherical; D = depth electrode; Y = yes; N = no.

### 3.2 Analysis: Network measures in SOZ and RA

#### 3.2.1 In‐degree

**Figure 3:**
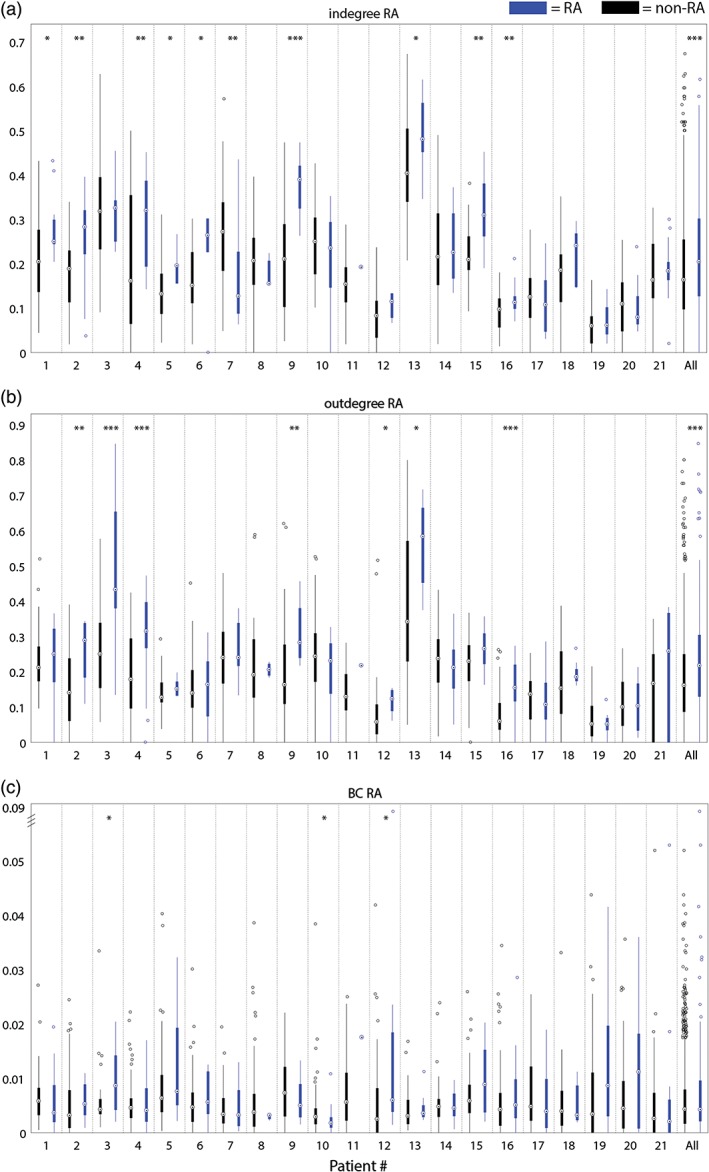
(a) The in‐degree in RA (blue) and in non‐RA (black); (b) the out‐degree in RA (blue) in non‐RA (black); (c) the betweenness centrality in RA (blue) and in non‐RA (black). Note that the *y*‐axis is broken to facilitate visibility of the low and wide distribution of BC‐values. * = *p* < .05, ** = *p* < .01, *** = *p* < .001 [Color figure can be viewed at http://wileyonlinelibrary.com]

In four patients (6, 9, 12, and 18), the in‐degree was higher in the SOZ compared to nodes in non‐SOZ (Figure 3a). These patients all became seizure free. In nine patients (1, 2, 4, 5, 6, 9, 13, 15, and 16), the in‐degree was higher in the RA, compared to non‐RA nodes. Eight of these patients (all except 13) were seizure free after surgery. In patient 7, the in‐degree was lower in the RA and SOZ, compared to non‐RA and non‐SOZ nodes. This patient was not seizure free after surgery.

When nodes of all patients were combined, the in‐degree was higher in both the SOZ (*p* = .01, data not shown) and RA (*p* < .001).

When we compared the group of patients with a good seizure outcome with the patients who were not seizure free (Figure 5a), the in‐degree was higher in the RA and SOZ compared with non‐epileptogenic tissue in the seizure free group (respectively, *p* < .001 and *p* = .002). In the not seizure free group, we did not find this difference in SOZ and non‐SOZ (*p* = .67), but we observed a lower in‐degree in RA compared to non‐RA (*p* = .006).

#### 3.2.2 Out‐degree

In three patients (3, 9, 12), the out‐degree was higher for nodes in the SOZ (data not shown) (Figure 3b). These patients were all seizure free after surgery. In seven patients (2, 3, 4, 9, 12, 13, and 16), the out‐degree was higher for nodes in the RA. Six of these patients (all except 13) were seizure free after surgery. At group level, the out‐degree was higher in both the SOZ (*p* < .001) and RA (*p* < .001).

When we compared the seizure‐free patients with the not seizure‐free patients (Figure 5a), the out‐degree was higher in the RA and SOZ compared to non‐epileptogenic tissue in seizure‐free patients (respectively, *p* < .001 and *p* = .004). Remarkably, the out‐degree was also higher in the SOZ compared to non‐SOZ in not seizure‐free patients (*p* = .02).

#### 3.2.3 Betweenness centrality

In patient 6, we found a higher betweenness centrality for the nodes in the SOZ (Figure 3c). In patient 3, 12, the betweenness centrality was higher in nodes in the RA. In patient 10, the betweenness centrality was higher in non‐RA nodes. At group level, we did not observe any differences. In seizure‐free patients (Figure 5a), we saw a trend toward significant lower betweenness centrality in RA than in non‐RA (*p* = .06). We did not see a difference in not seizure‐free patients.

### 3.3 Directionality of connections for each node

#### 3.3.1 Activating connections

**Figure 4:**
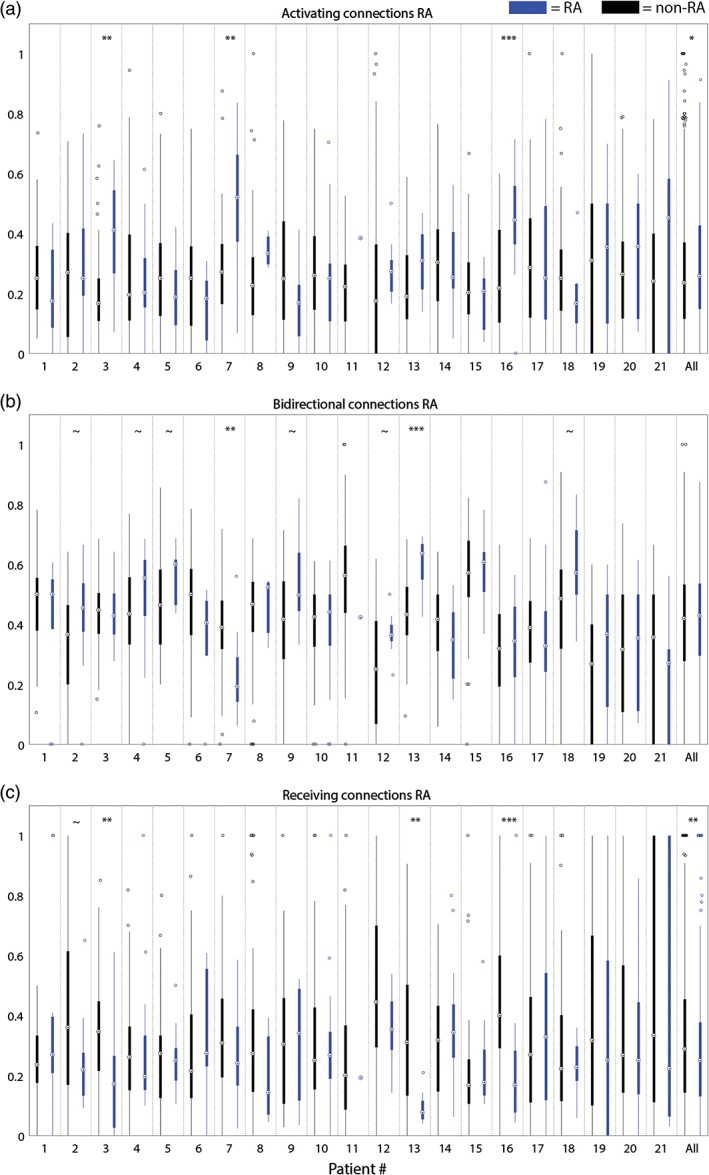
(a) Ratio of activating connections of all connections involving each node in the RA (blue) and non‐RA (black); (b) ratio of bidirectional connections of all connections involving each node in the RA (blue) and non‐RA (black); (c) ratio of receiving connections of all connections involving each node in the RA (blue) and non‐RA (black). ~ = *p* < .1, * = *p* < .05, ** = *p* < .01, *** = *p* < .001 [Color figure can be viewed at http://wileyonlinelibrary.com]

**Figure 5:**
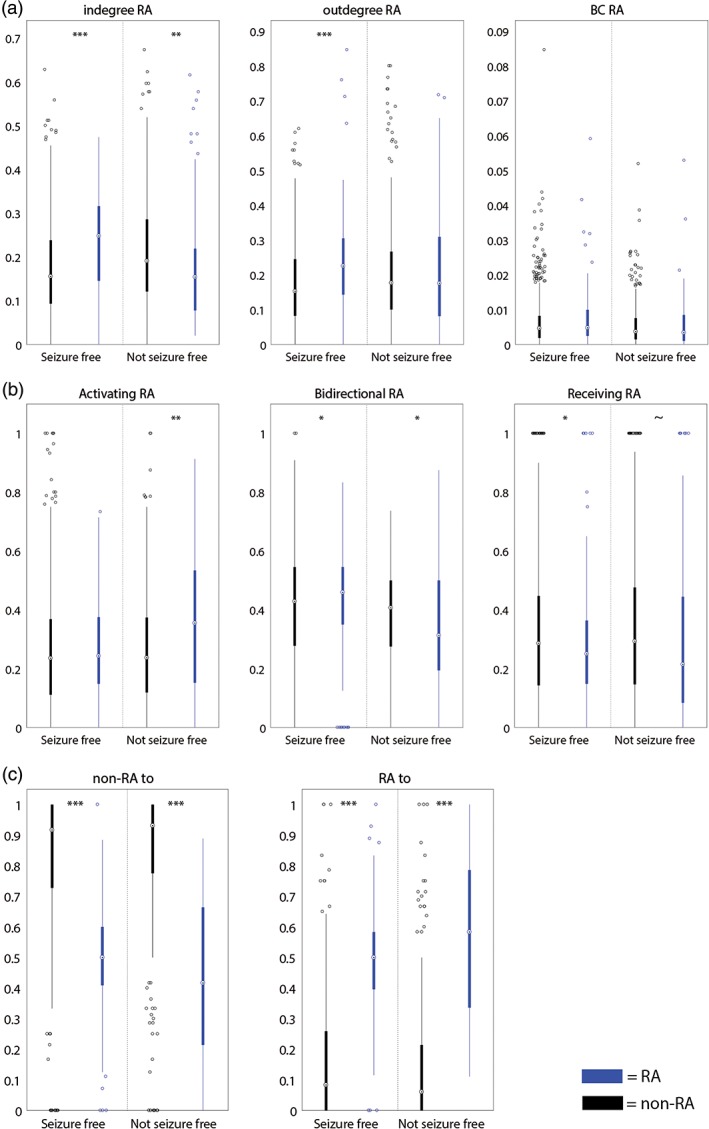
(a) The in‐degree, out‐degree, and betweenness centrality in the RA (blue) and non‐RA (black) in seizure‐free and not seizure‐free patients; (b) ratio of activating, bidirectional and receiving connections of all connections involving each node in the RA (blue) or non‐RA (black) for seizure‐free patients and not seizure‐free patients; (c) ratio of connections from (non‐)RA nodes to (non‐)RA nodes in both seizure‐free and not seizure‐free patients. In both seizure‐free and not seizure‐free patients, the ratio of connections from non‐RA nodes to non‐RA nodes is higher than to RA nodes. The ratio of RA nodes to RA nodes is higher than to non‐RA nodes. ~ = *p* < .1, * = *p* < .05, ** = *p* < .01, *** = *p* < .001 [Color figure can be viewed at http://wileyonlinelibrary.com]

In three patients (3, 7, and 16), the percentage of activating connections was higher in the RA than in non‐RA nodes (Figure 4a). In two patients (3 and 7), the same results were found in SOZ compared to non‐SOZ. At group level, a higher percentage of activating connections was found in the RA than in non‐RA nodes (*p* = .01). When comparing seizure‐free patients with not seizure‐free patients, we observed a higher percentage of activating connections in the RA than in non‐RA nodes in not seizure‐free patients (*p* = .001) (Figure 5b). A similar trend was observed in SOZ nodes in not seizure‐free patients (*p* = .06).

#### 3.3.2 Bidirectional connections

In one patient (13), the percentage of bidirectional connections was higher in the RA than in non‐RA nodes (Figure 4b). A similar trend (*p* < .1) was found in six other patients (2, 4, 5, 9, 12, and 18). In one patient (7), a lower percentage of bidirectional connections was found in both the RA and SOZ nodes compared to non‐epileptogenic nodes. At group level, no difference was found between the epileptogenic and non‐epileptogenic nodes. The percentage of bidirectional connections was lower in the RA in not seizure‐free patients (*p* = .03) and higher in the RA in seizure‐free patients (*p* = .04; Figure 5b).

#### 3.3.3 Receiving connections

In three patients (3, 13, and 16), the percentage of receiving connections was lower in the RA than in non‐RA nodes (Figure 4c). In patient 3, this was also found for the SOZ compared to non‐SOZ. At group level, the same result was observed for the RA (*p* = .01). In seizure‐free patients, a lower percentage of receiving connections was observed in the RA compared to non‐RA nodes (*p* = .05) (Figure 5b). A similar trend was observed in RA nodes in not seizure‐free patients (*p* = .10).

### 3.4 The destination of connections from in and outside epileptogenic tissue

In all but two patients (11 and 13), the ratio of non‐RA nodes with connections to non‐RA nodes was higher than to RA nodes. In all but three patients (6, 11, and 13), the ratio of RA nodes with connections to RA nodes was higher than to non‐RA nodes. The same results were visible in most of the patients when analyzing SOZ nodes, at group level, or seizure‐free and non‐seizure‐free patients separated (*p* < .001; Figure 5c).

## 4 DISCUSSION

### 4.1 Main findings

**Figure 6:**
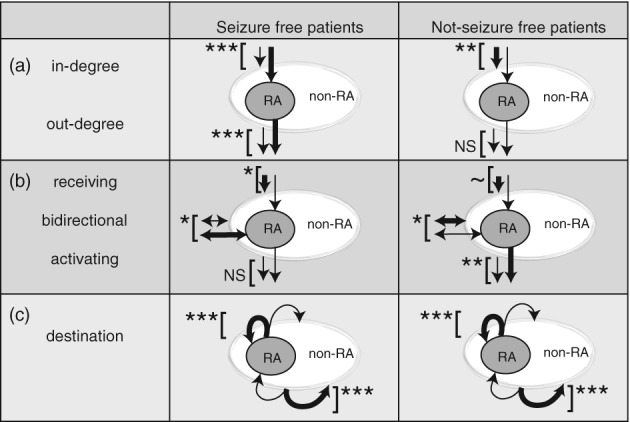
Summary of findings. (a) In‐degree and out‐degree in seizure‐free and not seizure‐free patients: The in‐degree was increased (displayed with a thick arrow toward the RA) in RA‐nodes compared to non‐RA nodes (displayed with a thin arrow toward the non‐RA) in seizure‐free patients. The opposite was found when comparing RA‐nodes with non‐RA nodes in not seizure‐free patients. The out‐degree was increased (displayed with a thick arrow originating from the RA) in RA‐nodes compared to non‐RA nodes (displayed with a thin arrow originating from the non‐RA) in seizure‐free patients. No difference in out‐degree was observed in not seizure‐free patients. (b) The directionality of connections in seizure‐free and not seizure‐free patients: The percentage of receiving connections (arrows toward the (non‐)RA‐areas) was decreased in RA‐nodes compared to non‐RA‐nodes in both seizure‐free and not seizure‐free patients. The percentage of bidirectional connections (arrows on both sides) was increased in the RA‐nodes compared to non‐RA nodes in seizure‐free patients. The opposite was found in not seizure‐free patients. The percentage of activating connections (arrows pointing from the [non‐RA]‐areas) was increased in RA‐nodes in not seizure‐free patients. No difference in percentage of activating connections was observed in seizure‐free patients. (c) The destination of connections from the RA or non‐RA nodes: In both seizure free and not seizure‐free patients, the ratio of connections from RA to RA‐nodes and non‐RA to non‐RA nodes was higher, suggesting an isolated epileptogenic area. NS = not significant, ~ = *p* < .1, * = *p* < .05, ** = *p* < .01, *** = *p* < .001

The in‐ and out‐degree were higher for nodes within epileptogenic tissue (SOZ and RA) in individual patients at group level and more so in seizure‐free patients. In not seizure‐free patients, the in‐degree was lower in the RA‐nodes. These results are summarized in Figure 6. In four patients (patients 2, 6, 15, and 16), the node with the highest in‐degree was located in the RA. In patient 20, the node with the highest in‐degree was located in the SOZ. This node was not resected during surgery and the patient was not seizure free after surgery. In none of the patients, the node with the highest out‐degree or betweenness centrality was located in the SOZ/RA.

Remarkably, patient 13, who did not become seizure free, also showed similar results for in‐ and out‐degree as other patients. This patient had a resection in the pericentral motor mouth area. Some tissue involved in seizure onset was not removed as the motor hand function was located there. After surgery, seizures changed to an onset with twitches in the hand. As part of the tissue involved in seizure onset was resected, this might explain the high in‐ and out‐degree in the epileptogenic tissue, although this patient was not seizure free after surgery. Furthermore, the difference in ratio between non‐RA nodes to (non‐)RA nodes and the difference in ratio between RA nodes to (non‐)RA was not significant, suggesting that the RA should have been larger to render this patient seizure free.

At group level, and when comparing seizure‐free and not seizure‐free patients, no difference was found in betweenness centrality inside or outside epileptogenic tissue.

The percentage of activating connections was higher in RA nodes in a few patients individually, at group level, and in not seizure‐free patients. In a few patients individually, at group level, and in seizure‐free patients, the percentage of bidirectional connections was higher in RA nodes. In a few patients individually, at group level and in seizure‐free patients, the percentage of receiving connections was lower in the RA nodes.

In most patients individually, at group level, and when comparing seizure‐free and not seizure‐free patients, the percentage of non‐RA nodes to non‐RA nodes was higher than non‐RA nodes to RA nodes and the percentage of RA nodes to RA nodes was higher than RA nodes to non‐RA nodes.

### 4.2 Implications

We found a high in‐degree and out‐degree in epileptogenic tissue. This is consistent with Mouthaan et al. (2015), who found a high count of ERs in the SOZ, which can be interpreted as a high in‐degree. Boido et al. (2014) reported that the epileptogenic zone can be identified by mapping bidirectionality features of ERs. A larger percentage of bidirectional and a lower percentage of receiving connections was observed in epileptogenic tissue (both RA/SOZ), as compared to non‐epileptogenic tissue. Hebbink et al. (2017) suggested a node that is driving the seizures is characterized by many connections originating from such a region, and only a few connections toward this region. Removal of this area may have a positive effect on seizure rate.

RA nodes had more connections to RA nodes than to non‐RA nodes, and non‐RA nodes had more connections to non‐RA nodes than to RA nodes. This result suggests that the RA is densely connected and that connections from non‐RA to RA are sparser.

In a recent review, Matsumoto, Kunieda, and Nair (2017) suggested that the amplitude of the ERs in epileptogenic and “normal” tissue is higher when stimuli are applied to the SOZ (Enatsu, Piao, et al., 2012), but the distribution of these ERs is not adapted in the epileptogenic network. In this study, we did not investigate the amplitude of an ER, but we found specific network properties of epileptogenic tissue.

### 4.3 Other research on functional networks

Most research on functional networks has focused on the ictal phase, or on the transition from the interictal to ictal phase. Several studies found highly interconnected nodes within epileptic networks in ictal scalp EEG (van Diessen et al., 2016; van Diessen, Hanemaaijer, et al., 2013), nodes with highest out‐degree in the RA in ictal SEEG (van Mierlo, Carrette, et al., 2013), or nodes with highest in‐ and out‐degree in the SOZ in patients with a good outcome (Courtens et al., 2016; Li et al., 2016). Khambhati et al. (2015) concluded that connections within the SOZ are the strongest. These studies describe the epileptogenic tissue as highly interconnected, resulting in high in‐ and out‐degree. This is in agreement with our findings of high in‐ and out‐degree in the epileptogenic tissue.

**Figure 7:**
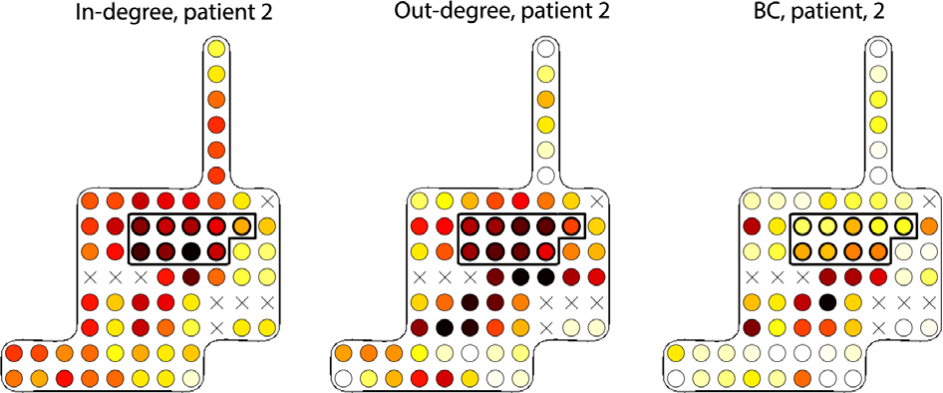
The in‐degree (left), out‐degree (middle), and betweenness centrality (right) in patient 2. The RA is enclosed by a thicker line (white = low, yellow = average, dark red = high). The in‐degree is high in the RA. There is a broad stripe of electrodes with a high out‐degree including the RA. Electrodes below the RA have a relatively high BC. X = electrodes excluded from analysis as these were not stimulated during SPES [Color figure can be viewed at http://wileyonlinelibrary.com]

We did not find betweenness centrality an indicator of epileptogenic tissue. Other studies only reported an increase in the betweenness centrality in the gamma band (Varotto, Tassi, Franceschetti, Spreafico, & Panzica, 2012; Wilke et al., 2011), or in a few seconds prior to seizure onset (Li et al., 2016). Geier, Bialonski, Elger, and Lehnertz (2015) found that the betweenness centrality in pre‐ictal ECoG (using cross‐correlation) was highest in brain regions neighboring the SOZ. This idea is supported by results for one of our patient, see the example in (Figure 7). For this patient, it is possible that a high in‐ and out‐degree in the SOZ led to a highly interconnected SOZ with only a small number of connections outside epileptogenic tissue.

### 4.4 Limitations

In this study, we used ECoG data, in which spatial sampling of the brain is limited to the location suspected of seizure onset and adjacent functional areas. Therefore, we are not able to extend our findings to large scale brain networks and to assert if a relationship between two nodes is direct, or indirect with an un‐sampled node in between. Furthermore, electrodes on the boundary of the grid might have shown fewer connections, as not all areas around them were sampled. We corrected for this by considering the number of stimulations in each electrode.

Another bias might have been that often the grids are placed in such a way that the presumed SOZ is located in the middle of the grid. This could have led to a higher in‐ and out‐degree because all areas around the grid‐centers are sampled. As it turned out, in 11 patients the SOZ/RA was actually located on a grid border, or on a strip with no sampled areas around the strip and in respectively 3 and 4 of these patients, the obvious differences in in‐ and out‐degree were still observed. We also calculated an average in‐degree and out‐degree for electrodes on edges, corners, strips, or middle of grids in 21 patients. When correcting for the mean number of connections in an electrode on a specific location, our results did not change.

There was some discrepancy between the RA and the SOZ. The clinically reported RA was larger than the clinically annotated SOZ in most patients. This was often due to anatomical lesions which were visible on MRI, and therefore resected even if outside the SOZ.

Similarly, in patients who continued to experience seizures after surgery, the RA may not have included all of the SOZ, and therefore, this might have affected our analysis.

The timing of SPES after implantation of the electrode grids varied among patients but was always at least 1 day after implantation, diminishing the possible effect of general anesthesia on network excitability.

A disadvantage of SPES is that although we evoke ERs during an interictal period, it is not clear how our effective network relates to interictal functional networks, or whether it is more similar to an ictal functional network. Future research investigating functional and effective networks in the same patient could give insight into this matter.

### 4.5 Future perspective

We found a high in‐ and out‐degree, a higher percentage of bidirectional connections, and a lower percentage of receiving connections in epileptogenic tissue, suggesting that the epileptogenic tissue is densely connected with itself. These characteristics suggest that analysis of ERs from SPES might indicate the location of epileptogenic tissue. Future studies should focus on analysis of ERs from SPES to localize epileptogenic tissue prospectively.

## 5 CONCLUSION

With this study, we have shown the differences in network properties between epileptogenic and normal tissue exist and may be found using effective SPES networks.
